# miR-204-5p promotes the adipogenic differentiation of human adipose-derived mesenchymal stem cells by modulating DVL3 expression and suppressing Wnt/β-catenin signaling

**DOI:** 10.3892/ijmm.2015.2160

**Published:** 2015-03-31

**Authors:** HONGHUI HE, KE CHEN, FANG WANG, LILING ZHAO, XINXING WAN, LINGHAO WANG, ZHAOHUI MO

**Affiliations:** Department of Endocrinology, Third Xiangya Hospital of Central South University, Changsha, Hunan 410013, P.R. China

**Keywords:** miR-204-5p, human adipose-derived mesenchymal stem cells, dishevelled segment polarity protein 3, Wnt/β-catenin

## Abstract

MicroRNAs (miRNAs or miRs) play an important regulatory role during adipogenesis, and have been studied extensively in this regard. Specifically, the switch between the differentiation of mesenchymal stem cells (MSCs) towards adipogenic vs. osteogenic lineages is regulated by miR-204 which controls the expression of Runx2. However, the association between miR-204-5p and the Wnt/β-catenin signaling pathway during adipogenesis has not yet been clarified. In the present study, we demonstrate that miR-204-5p regulates the *in vitro* adipogenesis of human adipose-derived mesenchymal stem cells (hADSCs). The level of miR-204-5p was shown to be gradually upregulated during adipocytic differentiation, together with the mRNA expression of the critical adipogenic transcription factors, cytidine-cytidine-adenosine-adenosine-thymidine (CCAAT) enhancer binding protein α (C/EBPα) and peroxisome proliferator-activated receptor γ (PPARγ), and the mature adipogenic marker, fatty acid binding protein 4 (FABP4). We further demonstrate that while the overexpression of miR-204-5p promotes adipogenesis, its knockdown causes the inhibition of this process. We then used bioinformatics tools and luciferase reporter assay to establish that dishevelled segment polarity protein 3 (DVL3), a key regulator of the Wnt/β-catenin signaling pathway, is a direct target of miR-204-5p. In addition, the overexpression of DVL3 led to an increase in β-catenin and cyclin D1 (CCND1) expression and, by contrast, the knockdown of DVL3 led to a decrease in the expression of β-catenin and CCND1. The knockdown of DVL3 significantly promoted adipogenesis. Finally, we demonstrated that the overexpression of miR-204-5p induced the downregulation of β-catenin and the canonical Wnt target gene, CCND1, in mature adipoctyes, while its knockdown led to their upregulation. Taken together, our data suggest that miR-204-5p regulates adipogenesis by controlling DVL3 expression and subsequently inhibiting the activation of the Wnt/β-catenin signaling pathway.

## Introduction

The root cause of obesity is an imbalance between energy intake and expenditure, a disturbance known to occur in conditions, such as type 2 diabetes, dyslipidemia, hypertension and other metabolic and cardiovascular diseases (1). At the cellular level, obesity is characterized by an increase in the size (hypertrophy) and number (hyperplasia) of adipoctyes. Hence, a complete understanding of the mechanisms regulating adipogenesis is essential and of utmost priority (2).

MicroRNAs (miRNAs or miRs) are small non-coding RNAs that negatively regulate target gene expression at the post-transcriptional level by binding to the 3′-untranslated region (UTR) of mature mRNAs, affecting their stability (3). miRNAs have been shown to play important regulatory roles in a variety of physiological processes, including cell differentiation, apoptosis and proliferation (4). Several miRNAs have been reported to either accelerate or inhibit adipogenesis, through the regulation of different signaling pathways (5). For example, miR-30 has been shown to inhibit the differentiation of mesenchymal stem cells (MSCs) into preadipocytes (6), and the miR-17-92 cluster has been shown to accelerate the clonal expansion of preadipocytes during early differentiation by targeting Rb2/p130 (7). Moreover, some miRNAs (miR-130, miR-27a/b, miR-378 and miR-519d) can directly regulate the levels of two critical transcription factors, peroxisome proliferator-activated receptor γ (PPARγ) and cytidine-cytidine-adenosine-adenosine-thymidine (CCAAT) enhancer binding protein α (C/EBPα), thus affecting the dynamics of adipogenesis (8–11). The signaling pathways mediated by transforming growth factor (TGF)-β and Wnt have been shown to negatively regulate adipogenesis. For example, the overexpression of miR-21 has been shown to downregulate the expression of TGF-β receptor 2 (TGFβR2), which in turn promotes adipogenesis, while the inhibition of this function of miR-21 induces an increase in TGFβR2 expression accompanied by reduced adipogenic differentiation (12). Three independent studies have reported that some miRNAs affect adipogenesis by regulating Wnt signaling. Specifically, miR-8 facilitates adipogenesis by targeting Wntless and CG32767 (13), while miR-344 has been shown to inhibit the adipocytic differentiation of 3T3-L1 cells by targeting glycogen synthase kinase-3β (GSK3β) (14). Qin *et al* (15) used a microRNA array to identify miRNas that regulate Wnt signaling either negatively (miR-210, miR-148a, miR-194 and miR-322) or positively (miR-344, miR-27 and miR-181) during adipogenesis in the 3T3-L1 cell line. In addition, the authors demonstrated that miR-210 promotes adipogenesis by targeting Tcf7l2, an important regulator of Wnt signaling (15).

The Wnt signaling pathway, due to its indispensable role in development, is highly conserved during evolution. Both canonical and non-canonical Wnt signaling have been shown to negatively regulate adipogenesis. In canonical Wnt signaling mediated by β-catenin, the ligands, Wnt10a and Wnt10b, bind to frizzled 1 (FZD1) receptors and low-density lipoprotein receptor-related protein (LRP)5/6 co-receptors leading to the phosphorylation of dishevelled segment polarity protein (DVL) and the degradation of Axin, followed by hypophosphorylation and the nuclear translocation of β-catenin. This in turn leads to the activation of downstream target genes, such as cyclin D1 (CCND1), accompanied by the inhibition of PPARγ and C/EBPα, causing a further decrease in adipogenesis (16).

Although miR-204 has been shown to promote the adipogenesis and inhibit the osteogenesis of human MSCs through the direct suppression of Runx2 (17), the effects of miR-204-5p on the activity of Wnt/β-catenin signaling and, consequently, on adipocytic differentiation remain unclear. In the present study, we demonstrate that miR-204-5p promotes the differentiation of human adipose-derived MSCs (hADSCs) into mature adipoctyes by suppressing the expression of DVL3, a positive regulator of Wnt/β-catenin signaling.

## Materials and methods

### Isolation and differentiation of hADSCs

hADSCs were obtained through the liposuction of subcutaneous adipose tissue, using previously described methods (18). The donors were required to provide signed informed consent prior to the commencement of the study, which was approved by the Human Ethics Review Committee of the Third Xiangya Hospital of the Central South University, Changsha, China. Briefly, 10 g of freshly isolated adipose tissue were washed with D-Hank’s buffer (Gibco/Life Technologies, Shanghai, China) thrice, dissected into 1x1 mm sections and digested with collagenase I (1 mg/ml; Sigma-Aldrich, St. Louis, MO, USA) for 1 h at 37°C. The dissociated tissue was then filtered through a 150-*μ*m nylon mesh and centrifuged at 1,000 rpm for 5 min. The precipitate was lysed further with red blood cell lysis buffer (Beyotime, Haimen, China) for 10 min, followed by centrifugation at 1,000 rpm for 10 min. The fraction of cells thus precipitated was washed with D-Hank’s buffer, resuspended in DMEM-F12 (Gibco/Life Technologies) containing 10% fetal bovine serum (FBS; Gibco/Life Technologies, Mulgrave, VIC, Australia) and cultured at 37°C in 5% CO_2_. The medium was replaced after 72 h; cells at passages 4–7 were used for further experiments. To induce the differentiation of the hADSCs, confluent hADSC cultures were grown in medium supplemented with 1 *μ*mol/l dexamethasone, 10 *μ*mol/l insulin, 0.5 mmol/l isobutylmethyl-xanthine (IBMX) and 200 *μ*mol/l indomethacin (all purchased from Sigma-Aldrich). The induction medium was replaced every 2 days.

### Transfection of hADSCs

Mimics, mimic-NC, agomir (cholesterol-conjugated 2′-O-methyl-modified mimics), agomiR-NC, antagomir and antagomiR-NC of miR-204-5p, were synthe-sized by Guangzhou RiboBio Co., Ltd. (Guangzhou, China). The miR-204-5p target site was predicted using online software, and found to be highly conserved among vertebrates. pVAX1- DVL3 and pVAX1-NC were purchased from Biovector Science Lab, Inc. (Beijing, China), and siRNA-DVL3 (si-DVL3) and siRNA-NC (si-NC) were prepared by Invitrogen (Carlsbad, CA, USA). The hADSCs were transfected with mimics, mimic-NC, agomir, agomiR-NC (100 nM), antagomir, antagomiR-NC (200 nM), pVAX1-DVL3, pVAX1-NC, siRNA-DVL3 or siRNA-NC using Lipofectamine 2000 (Invitrogen) according to the standard manufacturer-recommended protocol. At 2 days following transfection, cell differentiation was induced using the aforementioned protocol.

### Bioinformatics analysis

The miRNA targets were predicted using PicTar (http://pictar.org/), TargetScan (http://www.targetscan.org/vert_42/) and miRanda (http://www.microrna.org/microrna/).

### Luciferase reporter constructs and assay

The 3′-UTR sequence of human DVL-3 containing the seed target sequence of miR-204-5p was amplified by PCR and cloned into the pmiR- RB-REPORT™ (Guangzhou RiboBio Co., Ltd.) dual luciferase plasmid. The following primers were used: forward, 5′-CCGCTCGAGCCCAGTGAGTTCTTTGTGGATGTG-3′ and reverse, 5′-GAATGCGGCCGCTGTGTGCCAGGCACTGTGCTAG-3′. The *Xho*I and *Not*I (all provided by Invitrogen) restriction sites are underlined above. The mutation of this sequence from AAAGGGA to ACCGGGA was performed using the QuickChange Site-Directed Mutagenesis kit (Agilent Technologies, Edinburgh, UK).

The pmiR-RB-REPORT vector (50 ng) containing either wild-type or mutated human DVL3 3′-UTR was co-transfected into the 293T cells (obtained from Xiangya Cells Center of Central South University, Changsha, China) together with miR-204-5p mimics (100 nM) using Lipofectamine 2000 (Invitrogen). A non-target control mimic was used as a transfection control. Four replicates were prepared for each transfection experiment. Firefly and *Renilla* luciferase activities were measured using the Dual-Glo Luciferase assay system (Promega, Madison, WI, USA).

### Reverse transcription-quantitative PCR (RT-qPCR)

Total RNA was isolated using the TRIzol RNA extraction kit (Life Technologies, Carlsbad, CA, USA). Reverse transcription of miR-204 was carried out using a reverse transcription kit (Life Technologies), according to a protocol with specific instructions for miRNAs (Guangzhou RiboBio Co., Ltd.). Briefly, the reverse transcription reaction was carried out in a total volume of 11 *μ*l, which included 4 *μ*l (2 *μ*g) total RNA, 2 *μ*l specific reverse transcription primer (500 nM) and 5 *μ*l RNase-free water. This reaction mixture was first incubated at 70°C for 10 min, followed by incubation in ice for 2 min. We then centrifuged the tubes briefly (5 sec) to collect the entire volume, and then added 5 *μ*l 5X reverse transcription Buffer, 2 *μ*l dNTP mix (2.5 mM), 0.5 *μ*l RNase inhibitor (40 U/*μ*l), 0.5 *μ*l reverse transcriptase (200 U/*μ*l) and 6 *μ*l RNase-free water in a total reaction volume of 25 *μ*l. The reaction mixture was then incubated at 42°C for 60 min, followed by 70°C for 10 min. miR-204-5p primer was designed by Guangzhou RiboBio Co., Ltd. and quantitative PCR (qPCR) was performed using the QuantiTect SYBR- Green PCR Master Mix kit (Toyobo Co., Ltd., Osaka, Japan) and the Mastercycler ep realplex RT-PCR cycler (Eppendorf, Hamburger, Germany). The U6 small nuclear RNA was used as an endogenous normalization control. The relative quantification difference for gene expression between different groups was determined using the 2^−ΔΔCT^ method, and the sequences of the primers are listed in Table I.

### Western blot analysis

To extract total protein, the cells were first lysed in ice-cold radio immunoprecipitation assay (RIPA) lysis buffer (Beyotime). The total protein concentration in the lysates was determined by BCA assay (Beyotime). A total of 50 *μ*g protein were separated by 10% sodium dodecyl sulphate-polyacrylamide gel electrophoresis (SDS-PAGE) gel and transferred onto a PVDF membrane (Millipore, Boston, MA, USA). The membrane was blocked in blocking buffer containing 0.1% Tween-20 and 5% skimmed milk for 1 h, followed by incubation in primary antibody overnight at 4°C. The following day, the membranes were washed in blocking buffer, incubated with HRP-conjugated secondary antibodies (Proteintech, Wuhan, China) for 1 h at room temperature, washed again and developed using the ECL kit (Beyotime). The following primary antibodies were used: DVL3 (ab76081; Abcam, Cambridge, UK), β-catenin (51067-2-AP), C/EBPα (18311-1-AP), fatty acid binding protein 4 (FABP4; 12802-1-AP) and PPARγ (16643-1-AP; all provided by Proteintech), CCND1 (BM0771; Wuhan Boster Biological Technology, Ltd., Wuhan, China).

### Oil Red O staining

The cells were washed twice in PBS, fixed in 4% paraformaldhyde (PFA; Beyotime) for 30 min, washed twice with PBS and stained with freshly prepared Oil Red O (Beyotime) working solution (60% Oil Red O stock solution, composed of 0.5% Oil Red O dissolved in isopropanol and 40% H_2_O) for 20 min at room temperature. The cells were then washed twice with water, and the stained lipid droplets were observed and imaged under a microscope (TE2000-E; Nikon, Tokyo, Japan).

### Statistical analysis

All data are presented as the means ± SD. All statistical analyses were performed using SPSS 13.0 software (SPSS Inc., Chicago, IL, USA). Statistical comparisons between 2 groups were made using the unpaired Student’s t-test. Multiple groups were analyzed and compared using one-way analysis of variance (ANOVA). A P-value <0.05 was considered to indicate a statistically significant difference.

## Results

### miR-204-5p is upregulated and DVL3 is downregulated in hADSCs during adipogenic differentiation

**Figure 1:**
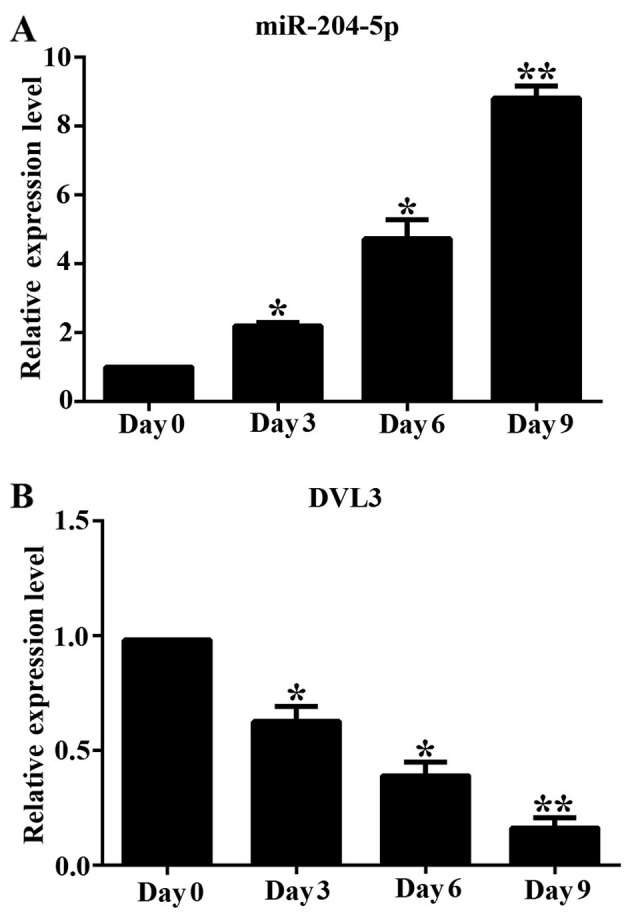
Upregulation of miR-204-5p and downregulation of dishevelled segment polarity protein 3 (DVL3) during the adipogenesis of human adipose-derived mesenchymal stem cells (hADSCs). Shown is the relative mRNA expression level of (A) miR-204-5p and (B) DVL3 at intervals of 3 days following a 9-day induction of adipogenic differentiation, as determined by RT-qPCR. Data collected from 3 independent experiments are represented as the means ± SD; ^*^P<0.05, ^**^P<0.01 in comparison to the relative expression levels on day 0.

We first determined the changes in the mRNA expression of miR-204-5p and DVL3 during the adipogenic differentiation of hADSCs *in vitro* at different time points (0, 3, 6 and 9 days). We measured the mRNA expression level of miR-204-5p and DVL3 by RT-qPCR, and found that miR-204-5p expression gradually increased during adipogenesis, by approximately 2.3-, 4.8- and 8.7-fold on days 3, 6 and 9, respectively, compared to the undifferentiated cells on day 0 (Fig. 1A). By contrast, the mRNA expression level of DVL3 decreased rapidly during adipogenesis, by approximately 37, 53 and 82% on days 3, 6 and 9, when compared to the undifferentiated cells on day 0 (Fig. 1B). This suggests that miR-204-5p plays an important physiological role during the differentiation of hADSCs into the adipocytic lineage, by regulating the levels of DVL3, a critical regulator of the Wnt/β-catenin pathway.

### miR-204-5p regulates the adipogenesis of hADSCs

**Figure 2:**
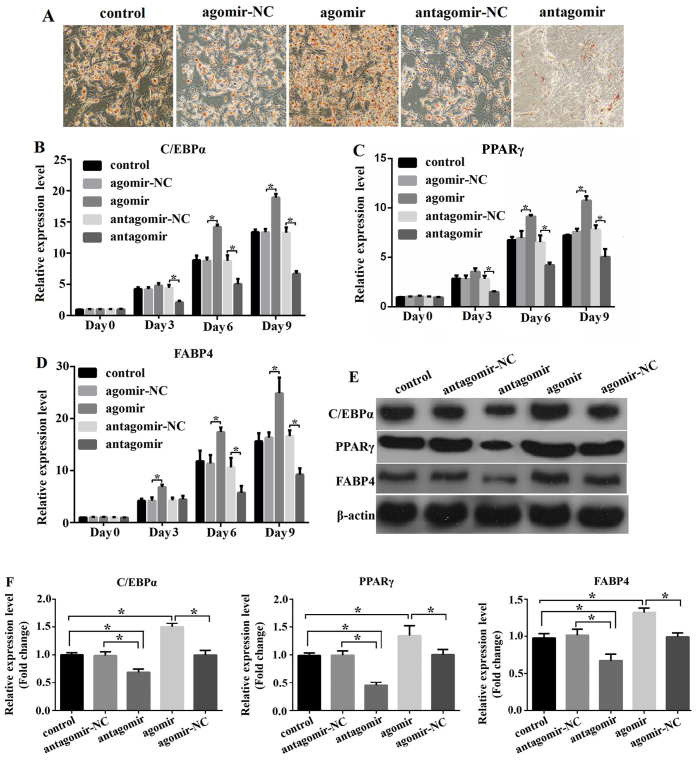
miR-204-5p regulates the adipogenic differentiation of human adipose-derived mesenchymal stem cells (hADSCs). hADSCs were transfected with miR-204-5p agomir, agomiR-NC (100 nM), antagomir or antagomiR-NC (200 nM). Untransfected hADSCs were used as controls. Differentiation was induced in cells in all groups for 9 days. (A) Shown are Oil Red O-stained hADSCs. (B-D) Also shown are the relative mRNA expression levels of the adipogenic genes, cytidine-cytidine-adenosine-adenosine-thymidine (CCAAT) enhancer binding protein α (C/EBPα), peroxisome proliferator-activated receptor γ (PPARγ) and fatty acid binding protein 4 (FABP4), at intervals of 3 days following a 9-day induction of differentiation. (E) Western blot analysis of the same genes and (F) densitometric analysis of the separated protein bands. ^*^P<0.05 compared to the control group (untransfected cells) at the same time point.

Although miR-204-5p is known to promote the differentiation of MSCs into mature adipoctyes by suppressing the expression of Runx2, to the very best of our knowledge, its effect on the adipogenic differentiation of hADSCs has not been demonstrated to date. To investigate this function of miR-204-5p, we transfected the hADSCs with miR-204-5p agomir, agomiR-NC, antagomir or antagomiR-NC, and then induced their differentiation for 9 days; the cells were then stained with Oil Red O to observe the lipid droplets in the differentiated cells. We found that the overexpression of miR-204-5p enhanced adipogenesis, resulting in increased numbers of stained cellular oil droplets in comparison to the control (untransfected cells) or in the differentiated hADSCs transfected with the negative control agomir (agomir-NC). Conversely, the knockdown of miR-204-5p significantly decreased the extent of adipogenesis (Fig. 2A). Subsequently, we examined the expression of the adipogenic marker genes, C/EBPα, PPARγ and FABP4, during normal adipogenesis, and found a gradual increase in the transcript and protein levels. Furthermore, the expression of all 3 genes markedly increased following the overexpression of miR-204-5p, while the knockdown of miR-204-5p led to a significant downregulation in the expression of these genes (Fig. 2B–D). Our results from RT-qPCR were consistent with those of western blot results for the differentiation period of 9 days. The overexpression of miR-204-5p in the hADSCs caused a significant upregulation in the protein levels of C/EBPα, PPARγ and FABP4, while its knockdown led to a significant decrease in the expression of these genes (Fig. 2E and F). Taken, our results suggest that miR-204-5p promotes the adipogenesis of hADSCs.

### DVL3 is a direct target of miR-204-5p

**Figure 3:**
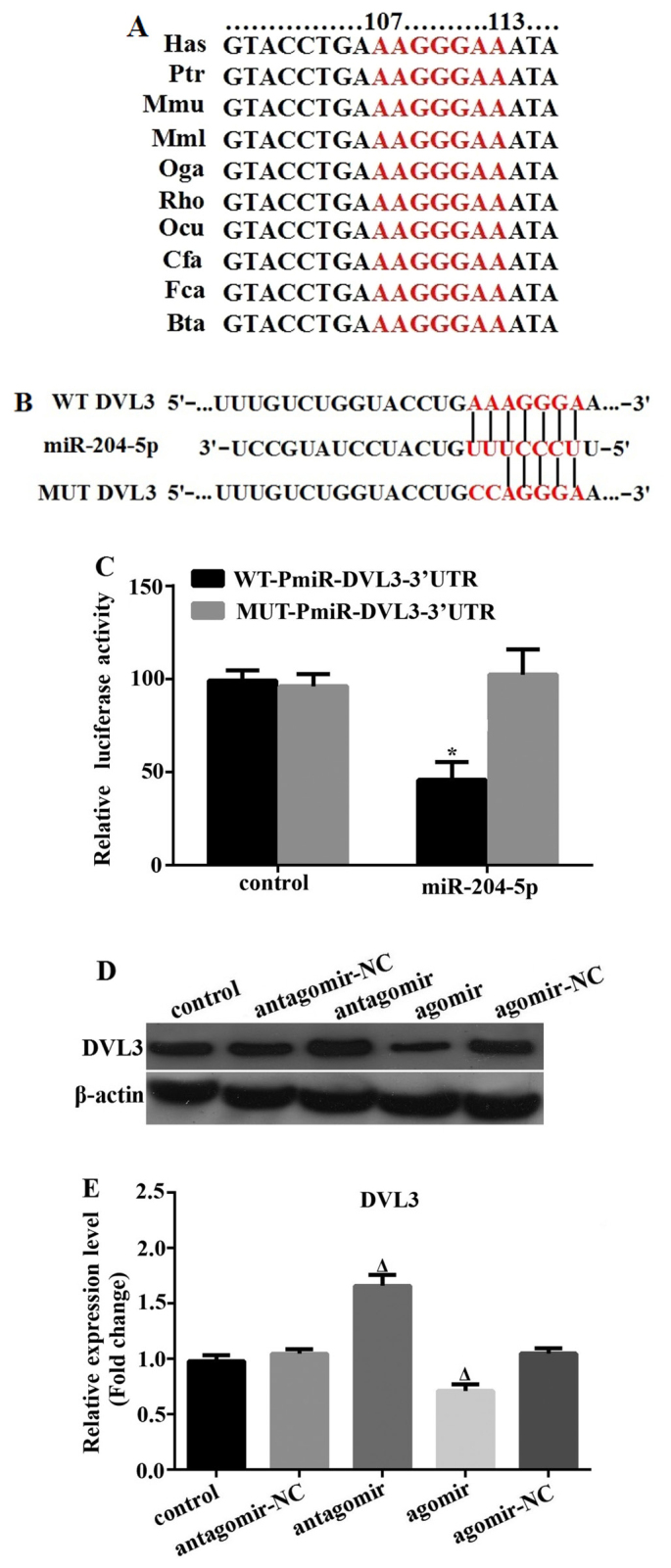
Disheveled segment polarity protein 3 (DVL3) is a direct target of miR-204-5p. Shown is the (A) miR-204-5p binding site (marked in red) in the 3′-UTR region of DVL3, and (B) the sequence of the binding regions in wild-type and mutated 3′-UTR. miR-204-5p seed matched region is highlighted in red. (C) Relative activity of *Renilla* luciferase, normalized to firefly luciferase, in 293T cells cotransfected with wild-type or mutated 3′-UTR luciferase reporter plasmid constructs and either miR-204-5p mimics or miR control mimics. (D) Protein expression of DVL3 following transfection of the human adipose-derived mesenchymal stem cells (hADSCs) with miR-204-5p antagomir, antagomiR-NC (200 nM), agomir or agomiR-NC (100 nM) for 72 h; and (E) densitometric analysis of separated protein bands. Data collected from 3 independent experiments are presented as the means ± SD. ^*^P<0.05, compared to 293T cells transfected with miR control mimics; ^Δ^P<0.05, compared to control (untransfected cells) or NC group.

In order to further explore the molecular pathway underlying the effects of miR-204-5p on the adipogenesis of hADSCs, we first identified potential target genes using the online prediction software, TargetScan, miRanda and PicTar. Specifically, DVL3 was found to possess the miR-204-5p target site in its 3′-UTR, and it was thus predicted, with a high score, to be a direct target by all 3 programs. In addition, the target site was found to be highly conserved among vertebrates (Fig. 3A). To confirm the predicted molecular interaction between miR-204-5p and the 3′-UTR of DVL3, we performed a standard dual-luciferase reporter assay using reporter plasmids carrying the wild-type or mutated 3′-UTR (of DVL3) seed target sequence (Fig. 3B). In comparison with the control miR mimic-transfected 293T cells, we found that co-transfection with the wild-type 3′-UTR luciferase plasmid and miR-204-5p resulted in a significant suppression of luciferase activity by 47%. By contrast, miR-204-5p had no effect on the luciferase activity of the mutated 3′-UTR reporter (Fig. 3C). Finally, we examined the protein expression level of DVL3 in the hADSCs following transfection with miR-204-5p antagomir or agomir for 72 h. In accordance with the above-mentioned findings, DVL3 expression was significantly upregulated following transfection with the miR-204-5p antagomir, and was markedly downregulated following transfection with the miR-204-5p agomir (Fig. 3D and E). These results clearly indicate that DVL3 is a direct target of miR-204-5p.

### DVL3 regulates adipogenesis through Wnt/β-catenin signaling

**Figure 4:**
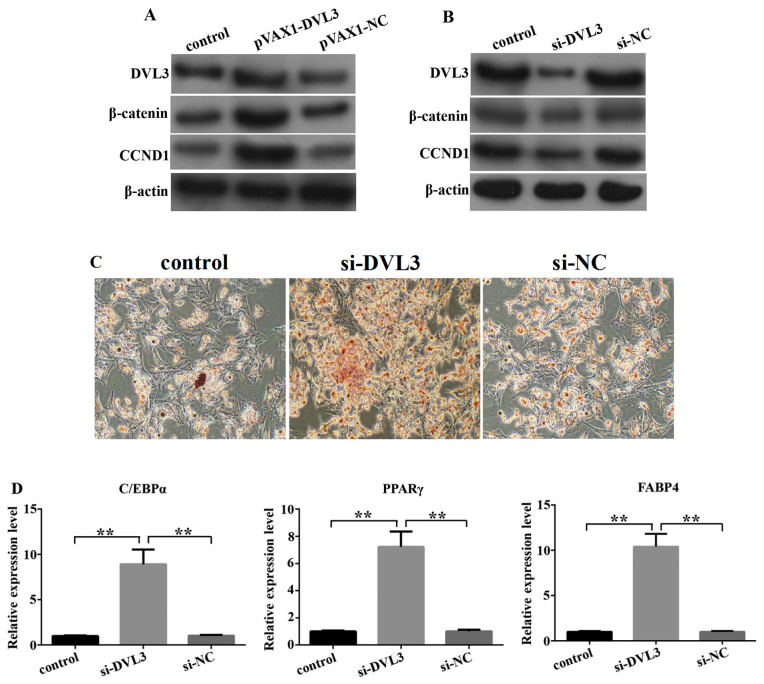
Disheveled segment polarity protein 3 (DVL3) regulates adipogenesis through Wnt/β-catenin signaling. (A and B) Protein expression of DVL3, β-catenin and cyclin D1 (CCND1) in human adipose-derived mesenchymal stem cells (hADSCs) transfected with pVAX1-DVL3, pVAX1-NC, si-DVL3 or si-NC for 72 h detected by western blot anlaysis. (C) Oil Red O staining of hADSCs following transfection with si-DVL3, si-NC for 48 h and induction of differentiation using a standard differentiation protocol for 9 days. (D) Relative mRNA levels of the adipogenic genes, cytidine-cytidine-adenosine-adenosine-thymidine (CCAAT) enhancer binding protein α (C/EBPα), peroxisome proliferator-activated receptor γ (PPARγ) and fatty acid binding protein 4 (FABP4), as determined by RT-qPCR.^**^P<0.01, compared to the control (untransfected cells) or si-NC group.

To determine whether DVL3 regulates adipogenesis, we first explored its effect on the activity of Wnt/β-catenin signaling by inducing its overexpression or knocking down its expression in hADSCs for a period of 72 h. We found that both β-catenin and CCND1 expression was upregulated in response to transfection with pVAX1-DVL3, and downregulated following transfection with siRNA-DVL3 (si-DVL3; Fig. 4A and B). Subsequently, we examined the changes in the extent of adipogenesis using Oil Red O staining in response to the knockdown of DVL3 in the hADSCs, following the induction of adipogenic differen-tation for 9 days. Our results indicated that transfection with si-DVL3 significantly promoted adipogenesis when compared with the control (untransfected cells) or the si-NC-transfected group. Furthermore, the expression of several markers of mature cells of the adipogenic lineage (C/EBPα, PPARγ and FABP4) was upregulated following the knockdown of DVL3, as determined by RT-qPCR (Fig. 4C and D). Our data suggest that DVL3 regulates the extent of adipogenesis by modulating the activity of Wnt/β-catenin signaling.

### miR-204-5p suppresses Wnt/β-catenin signaling, thus promoting adipogenesis

**Figure 5:**
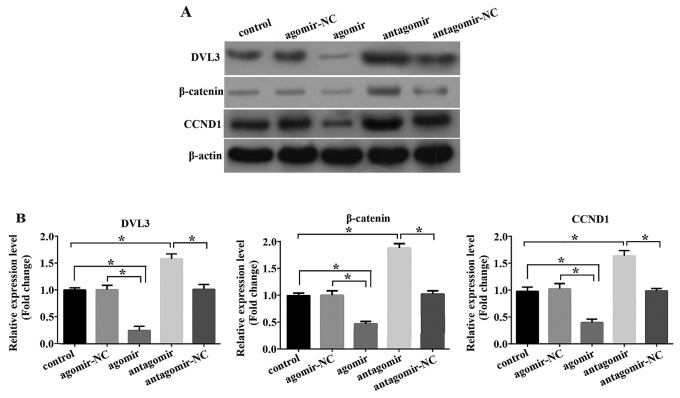
miR-204-5p regulates the activity of Wnt/β-catenin signaling in human adipose-derived mesenchymal stem cells (hADSCs). The differentiation of hADSCs transfected with miR-204-5p agomir, agomiR-NC (100 nM), antagomir or antagomiR-NC (200 nM) and non-transfected hADSCs (control) was induced for 9 days. Shown is the estimation of disheveled segment polarity protein 3 (DVL3), β-catenin and cyclin D1 (CCND1) expression by (A) western blot analysis and (B) densitometric analysis. ^*^P<0.05, compared to control (untransfected cells) or NC group.

DVL3 is a critical regulator of canonical Wnt/β-catenin signaling. Based on the above- mentioned results demonstrating that DVL3 is a direct target of miR-204-5p, we wished to determine whether this miRNA regulates the activity of the Wnt/β-catenin pathway. For this purpose, we transfected the hADSCs with either miR-204-5p agomir, agomiR-NC, antagomir or antagomiR-NC and 2 days later, adipogenic differentiation was induced for 9 days, and we then determined the relative activity of Wnt/β-catenin signaling by measuring the expression level of DVL3. As shown in Fig. 5A and B, the DVL3 protein levels were markedly decreased in the cells transfected with the miR-204-5p agomir, while transfection with the miR-204-5p antagomir led to a significant increase in DVL3 expression. Of note, the expression of β-catenin, the mediator of canonical Wnt signaling, and that of CCND1, a downstream target of this pathway, showed the same pattern of alteration as DVL3. Thnus, our results suggest that miR-204-5p suppresses canonical Wnt/β-catenin signaling, possibly by regulating DVL3 expression, which subsequently promotes adipogenesis.

## Discussion

The differentiation of MSCs into mature adipoctyes involves two main steps: i) the differentiation of multipotent MSCs into committed preadipocytes; and ii) the maturation of preadipocytes into adipoctyes through a molecular mechanism mediated by PPAR and C/EBP, two transcription factors known to regulate adipogenesis (19). miR-204 has been described as a ‘switch’ molecule that can control the fate choice between osteogenesis and adipogenesis. The overexpression of miR-204 has been shown to promote adipogenesis (17). However, little is known about its role in regulating Wnt/β-catenin signaling during adipocytic differentiation.

The role of miR-204 in regulating adipocytic pathways is unclear. A previous study demonstrated a downregulation in the expression level of miR-204 in the mature adipose tissue of C57BLJ6 mice fed a high-fat diet compared to those mice fed a standard diet (20). Another study demonstrated that the miR-204 levels were significantly upregulated in rat stromal vascular fraction (SVF) cells following adipocytic differentiation (21). Similar findings were reported by two other groups that studied C3H10T1/2 (22) and mesenchymal progenitor cell lines (17).

In the present study, we first examined the dynamics of miR-204-5p expression during adipogenesis in hADSCs, and found that its expression is gradually upregulated during this process. This result is consistent with the results of a previous study using SVF and C3H10T1/2 cells (22). We then demonstrated that the overexpression of miR-204-5p enhanced the adipogenesis of hADSCs, as illustrated by the significant increase in the quantity of lipid droplets and in the mRNA expression of PPARγ, C/EBPα and FABP4. Conversely, the downregulation of miR-204-5p suppressed adipogenic differentiation. Our data strongly suggest that miR-204-5p plays an important physiological role in regulating the adipogenesis of MSCs.

Wnt/β-catenin signaling has been shown to negatively regulate adipogenic differentiation in both MSCs and preadipocytes. The disruption of any component of this pathway significantly affects the generation of adipocytes. For example, as previously demonstrated in 3T3-L1 cells, the overexpression of Wnt1, Wnt10b or a GSK3β-mediated phosphorylation-defective form of β-catenin, inhibit adipogenesis (23). The constitutive expression of Axin or T-cell transcription factor 4 (TCF4) has been shown to induce spontaneous adipogenic differentiation (24). Similar observations have been made using MSCs (16). DVL is a positive regulator of Wnt/β-catenin signaling. Accordingly, the exposure of 3T3-L1 cells to anti-adipogenic compounds, such as 5-aminoimidazole-4-carboxamide-1-β-d-ribofuranoside (AICAR), epigallocatechin gallate (EGCG), platycodin D or 1,25-dihydroxyvitaminD3 [1,25(OH)_2_D_3_] has been shown to induce the upregulation of DVL (25–29). In the present study, we demonstrated that DVL3 expression progressively decreased during the differentiation of hADSCs, suggesting that the former may be a negative regulator of adipogenesis. Similar findings were reported in the studies by Lagathu *et al* (30) and Lee *et al* (25).

In order to further explore the mechanisms underlying the effects of miR-204-5p on adipocytic differentiation, we performed bioinformatics analysis and a dual luciferase reporter assay and verified that DVL3 is direct target of this miRNA. We then examined the direct role of DVL3 in regulating adipogenesis. Lee *et al* (31) performed a series of experiments through which they determined that compounds that inhibit adipogenesis, do so by upregulating components of Wnt/β-catenin signaling. For example, treatment with 200 *μ*M baicalin, a natural flavonoid compound extracted from *Suctellaria baicalensis*, was shown to significantly inhibit adipogenesis by downregulating the expression of PPARγ, C/EBPα and FABP4 in mature adipoctyes. In the control groups (0 *μ*M baicalin), the β-catenin and CCND1 mRNA levels were gradually downregulated during adipogenesis, while in the cells exposed to baicalin, these levels were markedly upregulated at different time points during differentiation. These findings were further verified using β-catenin-targeted siRNA (31). In addition, in this study, we demonstrated that the overexpression of DVL3 led to an increase in β-catenin and CCND1 expression, and conversely, the knockdown of DVL3 led to a decrease in β-catenin and CCND1 expression. In addition, the knockdown of DVL3 in hADSCs significantly promoted adipogenesis following induction for 9 days. Our findings indicate that DVL3 regulates the extent of adipogen-esis by modulating Wnt/β-catenin signaling. These findings were further corroborated by inducing the overexpression or knockdown of miR-204-5p, that led to alterations in the protein level of DVL3. To demonstrate the effect of miR-204-5p on the activity of Wnt/β-catenin signaling, we analyzed the expression of downstream effectors and targets of this pathway in response to the overexpression or knockdown of miR-204-5p in the hADSCs. We demonstrated that DVL3, β-catenin and CCND1 expression was downregulated and upregulated in mature adipoctyes, in response to transfection with the miR-204-5p agomir or antagomir, respectively.

In conclusion, the present study provides direct evidence that miR-204-5p induces the post-transcriptional silencing of DVL3, an important negative regulator of adipogenesis. We also demonstrate that miR-204-5p promotes adipogenesis by regulating the activity of canonical Wnt/β-catenin signaling in hADSCs. These findings suggest that DVL3 and miR-204-5p may serve as potential therapeutic targets for the treatment and management of obesity and other related metabolic disorders.

## Figures and Tables

**Table I:** Sequence information on the primers used for RT-qPCR.

Gene name	Sequences of primers
DVL3	F: 5′-ACAATGCCAAGCTACCATGCTTC-3′
	R: 5′-AGCTCCGATGGGTTATCAGCAC-3′
C/EBPα	F: 5′-TGGACAAGAACAGCAACGAG-3′
	R: 5′-TTGTCACTGGTCAGCTCCAG-3′
PPARγ	F: 5′-GAGAAGACTCAGCTCTAC-3′
	R: 5′-CAAGCATGAACTCCATAGTG-3′
FABP4	F: 5′-AGCACCATAACCTTAGATGGGG-3′
	R: 5′-CGTGGAAGTGACGCCTTTCA-3′
GAPDH	F: 5′-GGCTGAGAACGGGAAGCTTGTCAT-3′
	R: 5′-CAGCCTTCTCCATGGTGGTGAAGA-3′

DVL3, dishevelled segment polarity protein 3; C/EBPα, cytidine-cytidine-adenosine-adenosine-thymidine (CCAAT) enhancer binding protein α; PPARγ, peroxisome proliferator-activated receptor γ; FABP4, fatty acid binding protein 4; GAPDH, glyceraldehyde-3-phosphate dehydrogenase.
